# Global perspectives on autism acceptance, camouflaging behaviours and mental health in autism spectrum disorder: A registered report protocol

**DOI:** 10.1371/journal.pone.0261774

**Published:** 2021-12-30

**Authors:** Connor Tom Keating, Lydia Hickman, Philippine Geelhand, Toru Takahashi, Joan Leung, Bianca Schuster, Alicia Rybicki, Teresa Marie Girolamo, Elise Clin, Fanny Papastamou, Marie Belenger, Inge-Marie Eigsti, Jennifer Louise Cook, Hirotaka Kosaka, Rieko Osu, Yuko Okamoto, Sophie Sowden

**Affiliations:** 1 School of Psychology, University of Birmingham, Birmingham, United Kingdom; 2 ACTE (Autism in Context: Theory and Experiment) at LaDisco (Center for Linguistics Research) and ULB Neuroscience Institute, Université libre de Bruxelles, Brussels, Belgium; 3 Faculty of Human Sciences, Waseda University, Tokorozawa, Japan; 4 School of Psychology, University of Aukland, Aukland, New Zealand; 5 Department of Psychological Sciences, University of Connecticut, Storrs, Mansfield, Connecticut, United States of America; 6 Department of Neuropsychiatry, University of Fukui, Fukui, Japan; 7 Waseda Institue for Advanced Study, Waseda University, Tokyo, Japan; Radboud University, NETHERLANDS

## 1. Study information

### 1.1. Description (optional)

Autism spectrum disorder (hereafter ‘autism’) is a neurodevelopmental condition that is characterised by difficulties in social communication and restricted and repetitive interests [1]. Throughout life, autistic people (‘identity-first’ terminology is used throughout in line with the preferences of the autistic community [2] and guidance for avoiding ableist language [3]) experience a higher risk of psychiatric and mental health disorders [4] and elevated risks of premature mortality by nearly two decades than their non-autistic peers [5]. In a population-derived sample of young autistic people aged 10 to 14, 71% met criteria for at least one mental health disorder, with 41% having additional pychiatric diagnoses [6]. Similarly high rates of mental health problems have been found in other studies of autistic adolescents [7] and autistic adults [8–11]. Two of the most common psychiatric problems for autistic adults are depression and anxiety [12–15], with prevalence rates for depressive and anxiety disorders reaching as high as 47.1% and 54.0%, respectively [16]. It is also possible that prevalence rates of co-occurring psychiatric and mental health difficulties are underestimated, with many autistic individuals being subject to missed, delayed or inaccurate diagnoses [17] and healthcare needs which remain unmet in health systems modelled on the needs of neurotypical individuals [18].

For autistic individuals, mental health difficulties increase the likelihood of poorer long-term outcomes [19] including lower social and adaptive functioning [10], educational and employment difficulties [20, 21], poorer quality of life [22–24], and suicidality [25]. In order to support autistic people in achieving positive long-term outcomes, it is important to further understand the presentation of mental ill-health in autism and to determine *why* autistic individuals are at higher risk of mental health difficulties. This is crucial since the mental health of autistic individuals has been identified as a key priority for research across multiple stakeholder groups [26, 27].

There are many factors that could contribute to elevated mental health difficulties in autism. For example, since the prevalence of alexithymia (a subclinical condition characterised by difficulties identifying, expressing and differentiating emotions [28]), a transdiagnostic risk factor for numerous psychiatric conditions (e.g., depression, [29], and anxiety disorders, [30]) is higher in the autistic [49.93%] than the non-autistic population (4.89%; [31]), alexithymia may lead to increased risk of mental health difficulties in autism. Alternatively, Botha and Frost [32] advocate the extension of the minority stress model to explain the substantial health disparities between autistic and non-autistic individuals. The minority stress model posits that high stress burdens can negatively impact the mental and physical health of marginalised groups [33]. Such stress burden and resulting poorer health is frequently observed in minority groups including sexual, gender identity and ethnic minorities [34, 35], and minority stressors have recently been described to increase depressive symptoms in those with physical disabilities [36]. Examples of minority stressors include everyday discrimination, internalised stigma, and concealment. In line with this, the current study focuses on the impact of autism acceptance and camouflaging (which are minority stressors) on the mental health of autistic individuals (a marginalised group).

#### 1.1.1. Autism acceptance, camouflaging and mental health

Autism acceptance, as discussed here, can be defined as “an individual feeling accepted or appreciated as an autistic person, with autism positively recognised and accepted by others and the self as an integral part of that individual” [37]. The majority of autistic individuals in the UK feel that society does not accept [43%] or only sometimes accepts [48%] them as an autistic person (in total 91%; [37]). In spite of this, until recently, research had not directly examined autistic people’s experiences of autism acceptance and how this relates to mental health difficulties. The first study to do so confirmed an association between acceptance and mental health, finding that autism acceptance from external sources predicted depression and stress [37] and personal acceptance predicted depression. In addition, individuals who spontaneously reported camouflaging their autistic traits also reported higher symptoms of depression and fewer experiences of acceptance in the past week [37].

Camouflaging can be defined as “the use of strategies by autistic people to minimise the visibility of their autism in social situations” [38]. These strategies can involve compensation, whereby autistic individuals develop behaviours to help them manage social situations (e.g., learning how to use eye contact or developing scripts to help them navigate social interactions; [38, 39]), and masking, whereby autistic individuals aim to hide their autistic traits (e.g., deliberately suppressing stimming behaviours; [38]). Whilst the vast majority of autistic individuals report camouflaging to some degree [38], there are indications that camouflaging may be more common among women [40–48] and those higher in autistic traits [40, 49–51]. There is inconsistent evidence regarding whether camouflaging is associated with broader cognitive ability [40], with some studies finding that a higher level of education is associated with greater camouflaging [49], and others suggesting there is no association between Intelligence Quotient (IQ) and camouflaging [38, 42, 52] or executive functioning and camouflaging [48].

However, a burgeoning literature suggests that camouflaging may be a risk factor for depression and anxiety in autism [25, 37, 53–57], irrespective of gender [58]. Interestingly, Cage and colleagues [37] suggest that camouflaging may mediate the relationship between social stressors (e.g., lack of autism acceptance, bullying) and anxiety and depression. That is, those who experience more social stressors (such as a lack of autism acceptance) are more likely to camouflage their autistic traits, which in turn results in higher levels of anxiety and depression. However, a recent study found that whilst autism-related stigma was associated with lower wellbeing, camouflaging did not mediate this relationship (despite camouflaging being positively associated with autism-related stigma; [59]). Future research should verify whether camouflaging mediates the relationship between autism acceptance/stigma and anxiety and depression more specifically.

It is important to note that the terms we use to refer to camouflaging vary across the globe [60]. In Japan, the term ‘over-adaptation’ is used to describe excessive efforts made by people (i.e., society more broadly) to meet external demands and expectations, even if this means forcibly suppressing their internal needs [61]. In recent years, this term has been applied to autism to describe the substantial efforts made by autistic people to fit into an environment as a result of differences in interpersonal and social behaviour [62–64]. As such, this concept is highly similar to the construct of camouflaging (see [61] for a discussion around the similarities and differences between the constructs of camouflaging and over-adaptation in autism). Importantly, there is anecdotal evidence that over-adapatation is associated with poorer mental health in autistic individuals in Japan [65]. However, to date no empirical studies have tested this relationship [61], and therefore further study of over-adaptation and camouflaging in Japan is necessary.

#### 1.1.2. Cross-cultural perspectives on autism acceptance, camouflaging and mental health

Culture can be defined as “a set of basic assumptions and values, orientations to life, beliefs, policies, procedures and behavioural conventions that are shared by a group of people” [66]. Unsurprisingly, across different cultures there can be large variation in these beliefs, attitudes and behaviours. In the case of autism, we see cross-cultural variation in attitudes towards autistic people: previous research has found that *non-autistic* college students in the US reported lower levels of autism-related stigma than their Lebanese [67, 68] and Japanese [69] counterparts. Whilst these studies demonstrate that there may be variation in levels of autism stigmatisation among *non-autistic* individuals across cultures, studies have not yet compared *autistic* individuals’ experiences of autism acceptance across different cultures. Given that levels of stigma reported by non-autistic individuals vary cross-culturally, it is plausible that experience of acceptance from the perspective of autistic individuals themselves also vary across cultures. Such cross-cultural differences may exist in both external acceptance (as experiencing stigma may result in autistic individuals feeling less accepted by others) and personal acceptance (as stigma can become internalised resulting in decreased personal acceptance or ‘self-stigma’; [54, 70, 71]).

As Lai and colleagues [72] highlight, it is paramount to determine the levels of camouflaging in the autistic population. Currently, it is unknown how many undiagnosed autistic adults exist [73]. However, it is thought that there are more undiagnosed autistic individuals in some areas of the world than others [74]. Investigating the rates of camouflaging across cultures will help us to work towards determining the true prevalence of autism, and its sex/gender ratio, across the globe. To our knowledge, studies have not yet explored camouflaging in autistic populations outside of the UK (e.g., [58]) and USA (e.g., [75]), though there are anecdotal reports of over-adaptation in Japan. Rather than assuming levels of camouflaging in the UK and USA reflect those globally, researchers should collaborate internationally to compare camouflaging behaviours cross-culturally. Cross-cultural perspectives could have great utility for understanding the mechanisms behind camouflaging, as the social environments in which autistic individuals aim to adapt to vary across cultures. For example, by determining whether the rates of camouflaging are higher within certain cultures, we can work towards identifying socio-cultural factors that may contribute to camouflaging. This is important as there is a consensus among researchers that ‘there is much to be learnt about mechanisms’ [72].

Whilst no research has compared camouflaging across cultures, it seems likely that rates of camouflaging will vary between cultures for a number of reasons. Firstly, in collectivist cultures (which prioritise community interdependence and shared group norms and values; [76]), there may be more pressure relative to individualistic cultures (which prioritise independence and individualism; [76]) to camouflage one’s autistic traits in order to minimise deviation from shared group norms. Thus, those in collectivist cultures may alter their behaviour or hide their autistic traits in order to avoid shame and fit in [62]. In contrast, in regions where there are more discussions about neurodiversity- both as a movement and as an ideology (e.g., the United States, Canada, and the United Kingdom; see [77])- individuals may feel less pressured to camouflage their autistic traits. Finally, since autism-related stigma varies across cultures [67–69] and reduced autism acceptance is associated with increased camouflaging [34, 61], levels of camouflaging may also differ cross-culturally.

Moreover, to the best of our knowledge, studies have not yet compared the level of mental health difficulties in cross-cultural samples of autistic individuals. Like camouflaging, there are reasons to believe there is cross-cultural variation in the level of these difficulties. Firstly, since autism acceptance differs across cultures [67–69], the level of mental health difficulties may also vary because a lack of autism acceptance or stigma is associated with poorer mental health [32, 37]. Secondly, since beliefs about mental health vary across cultures [78], and these beliefs can affect willingness to seek and adhere to treatment [79], there may be cross-cultural differences in levels of mental health difficulties. Finally, given that accessibility of mental health services also differs across cultures [80], individuals from certain regions may be unable to get the help they need, thus leading to poorer mental health. Research is necessary to explore whether the levels of mental health difficulties experienced by autistic individuals vary across cultures.

#### 1.1.3. The current study

In the current study, we aim to replicate the regression analyses from previous research that found a) depression was predicted by external and personal acceptance, and stress was predicted by external acceptance [37], b) camouflaging was predicted by stigma [59], and c) anxiety was predicted by camouflaging [58] (whilst controlling for relevant participant characteristics, e.g., age, gender, etc.) in a more representative sample of autistic adults. In addition, we aim to compare autistic individuals’ experiences of autism acceptance, camouflaging and mental health across multiple countries: Australia, Belgium, Canada, Japan, South Africa, the United Kingdom, and the United States. Importantly, exploring these factors across cultures has the potential to a) elucidate the potential routes to greater autism acceptance, reduced need for camouflaging behaviours and improved mental health outcomes, b) identify ‘priority regions’ for anti-stigma interventions (like those in Ranson and Byrne, [81] and Gillespie-Lynch et al., [82]), and c) highlight areas where greater support for mental health difficulties is needed. In addition, since acceptance is likely to vary across cultures, we are employing an experimental design which naturally maximises variation in autism acceptance, thus giving us the ability to more sensitively assess the impact of autism acceptance on camouflaging and mental health respectively.

In order to fulfil our aims, participants will complete a number of questionnaires including Autism Acceptance Questions [37], the Camouflaging Autistic Traits Questionnaire [51], and the 21-item Depression, Anxiety and Stress Scale (DASS-21; [83]). Participants will also provide demographic information (e.g., age, sex assigned at birth, gender identity, sexual identity, ethnicities, country of birth, country of residence, years lived in country of residence, number of siblings, level of education, income, autism diagnosis, person/team that gave them their diagnosis, age of autism diagnosis, co-occuring diagnoses, how verbal they would consider themselves now, and how verbal they were as a child).

### 1.2. Hypotheses (required)

#### 1.2.1. Replicating the regression analyses from previous research

First, we will conduct regression analyses on our data as a whole (i.e., participant data will be analysed as one large and diverse group) to replicate the findings from previous studies [37, 58, 59] in a more diverse sample. In line with Cage and colleagues [37], we predict that external acceptance and personal acceptance will contribute to depression scores, after controlling for age, gender, age of diagnosis, anxiety scores and stress scores. In addition, we predict that external acceptance will contribute to stress scores, after controlling for age, gender, age of diagnosis, anxiety scores and depression scores. As was the case in Perry et al., [59], we hypothesise that autism acceptance will predict camouflaging scores after controlling for age, gender, age of diagnosis, and autistic traits. In line with Hull et al., [58] we predict that camouflaging will contribute to anxiety scores after controlling for age and autistic traits.

#### 1.2.2. Comparing levels of autism acceptance, camouflaging and mental health across cultures

Finally, we will separate participants into groups based on their country and use a between-subjects multivariate analysis of variance (MANOVA) to explore participants’ experiences of autism acceptance, camouflaging, and levels of depression, anxiety and stress (dependent variables) across these countries (independent variable). We predict that there will be differences in the level of autism acceptance across countries. In addition, we aim to explore whether there are any differences in the level of camouflaging and mental health difficulties across countries.

## 2. Design plan

*In this section, you will be asked to describe the overall design of your study. Remember that this research plan is designed to register a single study, so if you have multiple experimental designs, please complete a separate preregistration*.

### 2.1. Study type (required)

Observational Study—Data is collected from study subjects that are not randomly assigned to a treatment. This includes surveys, natural experiments and regression discontinuity designs.

### 2.2. Blinding (required)

No blinding is involved in this study.

### 2.3. Study design (required)

This study employs a between-subjects design, where we will be comparing participants experiences of personal acceptance, external acceptance, camouflaging, depression, anxiety and stress across cultures.

### 2.4. Randomization (optional)

No randomization is involved in this study.

## 3. Sampling plan

*In this section we’ll ask you to describe how you plan to collect samples, as well as the number of samples you plan to collect and your rationale for this decision. Please keep in mind that the data described in this section should be the actual data used for analysis, so if you are using a subset of a larger dataset, please describe the subset that will actually be used in your study*.

### 3.1. Existing data (required)

Registration prior to creation of data: As of the date of submission of this research plan for preregistration, the data have not yet been collected, created, or realised.

### 3.2. Data collection procedures (required)

Participants will be recruited via an existing international autism research database kept by the U21 Autism Research Network. The database was set up as part of a collaboration between seven autism research groups across 4 continents and 7 countries (Australia, Belgium, Hong Kong, Japan, New Zealand, UK, and USA). The aim of this initiative was to diversify samples recruited for autism research and to generate opportunities to answer questions relevant to the global autistic community. In this particular study, we will be recruiting participants from our sites in Belgium, Japan, UK and USA. In addition, we will recruit participants via Prolific, thus allowing us to recruit participants from Australia, Canada, and South Africa (amongst others), and via social media. All participants will provide informed consent. This study has been approved by the Science, Technology, Engineering and Mathematics (STEM) committee at the University of Birmingham (ERN_16-0281AP9D).

### 3.3. Sample size (required)

The final sample in this study will comprise at least 286 autistic adults (aged over 18) from across the globe.

### 3.4. Sample size rationale (optional)

#### 3.4.1. Replicating the regression analyses from previous research

**Fig 1:**
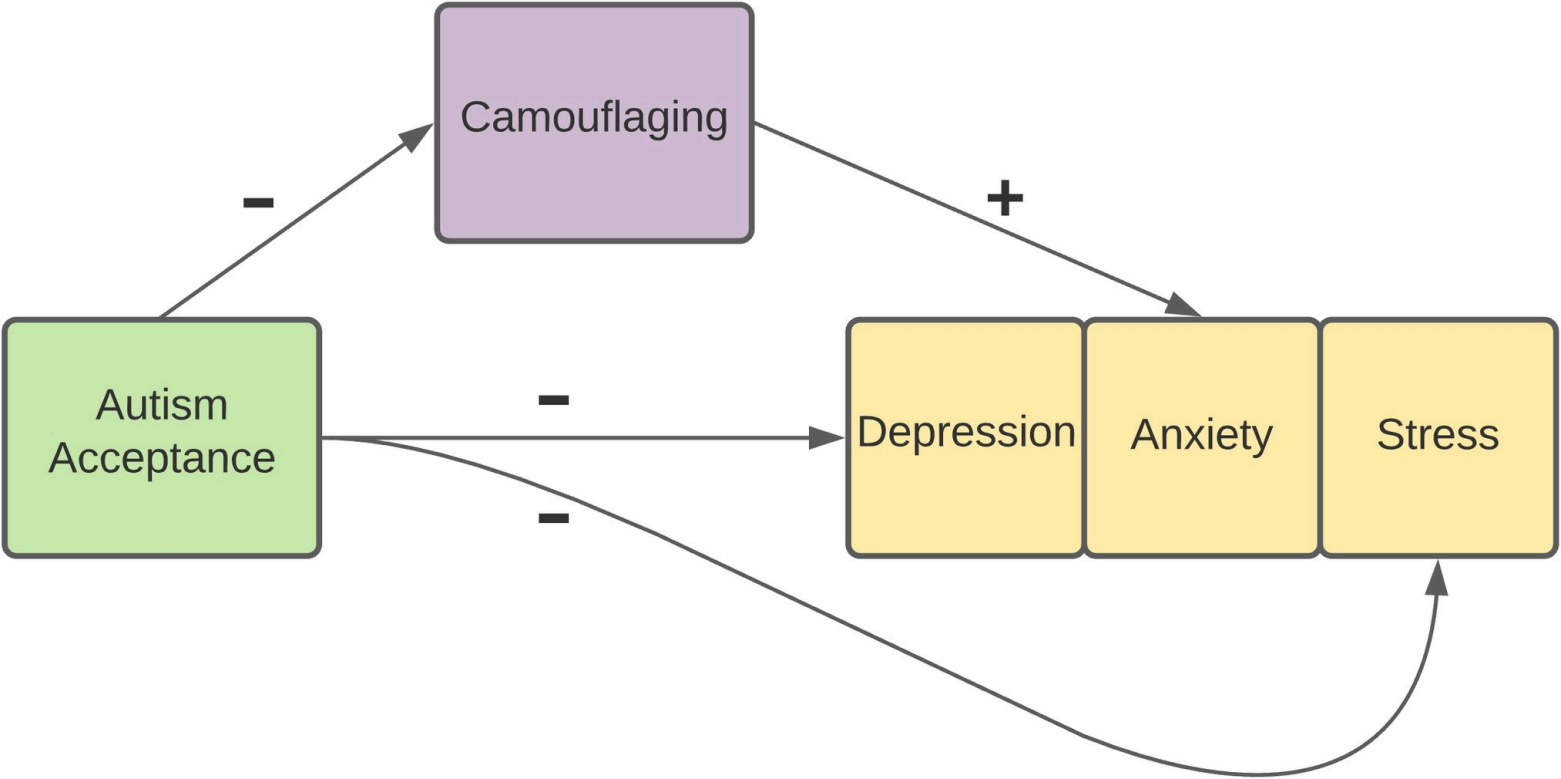
A diagram showing the relationships that we will test in the current study. The plus (+) indicates a positive relationship between the variables and the minus (-) indicates a negative relationship.

In our regression analyses, we will include data for all participants that complete the study in one group (regardless of country of residence) to replicate the findings from previous studies [37, 58, 59] in a large and cross-cultural sample of autistic adults. The chosen sample size for these analyses is based on a series of sample size calculations conducted using G*Power [84], which focus on replicating the regression analyses of interest from Cage et al., [37], Perry et al., [59] and Hull et al., [58]. As a whole, we aim to verify whether experiencing a lack of autism acceptance predicts greater camouflaging and heightened depression and stress, and whether increased camouflaging predicts elevated anxiety (see Fig 1). Further details about the specific analyses can be seen below.

Please note that for the following sample size calculations, we used an Bonferroni-adjusted alpha level of 0.0125 to account for multiple testing (4 regression analyses), and a power level of 90% (thereby giving us higher power than is standard in the field [85, 86]). To replicate the finding from Cage et al., that external acceptance and personal acceptance account for 17.5% variance explained in depression scores after controlling for age, gender, age of diagnosis, anxiety and stress (f^2^ = 0.37), 51 participants are required. In order to replicate the finding that external acceptance and personal acceptance account for 3% variance explained in stress scores after controlling for age, gender, age of diagnosis, anxiety and stress, 246 participants are required. To replicate the finding from Perry et al., that stigma significantly predicts camouflaging (f^2^ = 0.069) after controlling for age, gender, age of diagnosis, and autistic traits, 211 participants are required. Finally, in order to replicate the finding that anxiety accounts for 4% variance explained in camouflaging scores after controlling for age and autistic traits, 286 participants are required. Therefore, the final sample will comprise at least 286 participants (with full datasets).

#### 3.4.2. Comparing autism acceptance, camouflaging and mental health across cultures

In the analyses where we will compare participants’ experiences of autism acceptance, camouflaging, and levels of depression, anxiety and stress across countries, we will only include data from countries that have at least 30 participants (and therefore we will have sufficient data to draw comparisons between these multiple groups). We selected this cut-off as Sekaran [87] highlights that “where samples are to be broken into sub-samples…a minimum sample size of 30 for each category is necessary”. Importantly, Sekaran [87] also highlights that “in multivariate research…the sample size should be several times (preferably 10 times or more) as large as the number of variables in this study”. By recruiting at least 286 participants to our study, our sample is over 47 times larger than the number of variables in our multivariate analysis (6 variables: external acceptance, personal acceptance, camouflaging, depression, anxiety and stress), is as large as necessary based on our power analyses (largest sample size generated = 286), and would facilitate comparisons between participants in up to 9 countries.

## 4. Variables

*In this section you can describe all variables (both manipulated and measured variables) that will later be used in your confirmatory analysis plan. In your analysis plan, you will have the opportunity to describe how each variable will be used. If you have variables which you are measuring for exploratory analyses, you are not required to list them, though you are permitted to do so*.

### 4.1. Manipulated variables (optional)

Group membership–The autistic participants will be divided into groups based on the country in which they live (Australia, Belgium, Canada, Japan, etc.) in our Multivariate Analysis of Variance (MANOVA).

### 4.2. Measured variables (required)

#### 4.2.1. Demographic information

Participants will be asked a number of demographic questions to establish the nature of the sample. These will include questions about a) age, b) sex assigned at birth, c) gender identity, d) sexual identity, e) ethnicities, f) country of birth, g) country of residence, h) years lived in country of residence, i) level of education, j) how many siblings they have, k) income, l) co-occuring diagnoses m) how verbal they would consider themselves now, and n) how verbal they were as a child (as recommended by Botha, Hanlon and Williams [88]). Participants will also be asked to confirm whether or not they have an autism diagnosis, which diagnosis they received (e.g., Autism vs. Autism Spectrum Disorder), who gave them their diagnosis (e.g., multidisciplinary team, doctor, clinical psychologist, etc.), and finally the age they received that diagnosis. Those without a formal diagnosis will not be eligible to participate in the study.

#### 4.2.2. Autism acceptance

To measure perceptions of autism acceptance, we will use the six self-report autism acceptance questions outlined in Cage et al., [37]. First, in order to obtain a categorical response for acceptance, participants will be asked whether they feel that society (which will be specified as the general public, made up of people who do not personally know them) generally accepted them, with “yes”, “no”, “sometimes” and “prefer not to say” as response options. Second, participants will be asked to respond to the statement “over the past week, I have felt accepted by society as an autistic person/ person with autism”, on a five-point Likert scale from “strongly agree” to “strongly disagree”. Next, to measure perceptions of autism acceptance from different sources, participants will also be asked: “how accepted by society do you feel as an autistic person?”; “how accepted by your family and friends do you feel as an autistic person?”; and “how much have you personally accepted yourself as an autistic person?”. They will report their responses on a scale from zero, “not at all”, to ten, “completely”. Finally, in order to obtain qualitative responses, participants will have the opportunity to “tell us more about experiences of acceptance or non-acceptance” in an open text box. The English version of this set of autism acceptance questions has acceptable internal consistency (Cronbach’s α = 0.64) and good validity [37].

#### 4.2.3. Camouflaging of autistic traits

To measure the extent to which participants camouflage their autistic traits, we will use the Camouflaging Autistic Traits Questionnaire (CAT-Q; [51]). The CAT-Q comprises 25 items (e.g., “In social situations, I feel like I’m pretending to be normal”), rated on a seven-point Likert scale (ranging from one, “Strongly Disagree” to seven, “Strongly Agree”). Total scores on the CAT-Q can range from 25–175, with higher scores reflecting greater camouflaging. This self-report questionnaire assesses three different domains relevant to camouflaging: compensation (strategies used to compensate for socio-communicative difficulties, e.g., “I have spent time learning social skills from television shows and films, and try to use these in my interactions”); masking (strategies used to present a non-autistic or less autistic persona to others, e.g., “I monitor my body language or facial expressions so that I appear interested by the person I am interacting with”), and assimilation (strategies used to fit in to uncomfortable social situations, e.g., “I have to force myself to interact with people when I am in social situations”). The English version of the CAT-Q has strong psychometric properties, including internal consistency (Cronbach’s α = 0.94) and test-retest reliability (Pearson’s r = 0.77; [51]).

#### 4.2.4. Depression, anxiety and stress

To measure levels of depression, anxiety and stress, we will use the Depression, Anxiety and Stress Scale (DASS-21; [83]). In this 21-item questionnaire, participants judge whether certain statements (e.g., “I found it hard to wind down”) could be applied to their life over the past week on a scale from one to four (1 = “did not apply to me at all; 2 = “applied to me some of the time”; 3 = “applied to me a considerable degree”; 4 = “applied to me very much or most of the time”). The 21 items can be reduced to 7 items each for the depression, anxiety and stress subscales. Total scores for each subscale are calculated and multiplied by two (meaning that scores for each subscale range from 14 to 42), with higher scores representing higher depression, anxiety and stress. The DASS-21 has been previously used with autistic participants (e.g., [37, 89]) and the English [37, 89], French [90], and Japanese [91] versions have good internal consistency and factor validity.

#### 4.2.5. Questionnaires to facilitate our analyses in which we control for other participant characteristics

The following questionnaires are included to facilitiate a) the exploration of the relationships found previously between autistic traits and camouflaging, and alexithymic traits and mental health, and b) our analyses in which we control for these participant characteristics (see Exploratory Analyses).

#### 4.2.6. Autistic traits

The autistic traits of all participants will be assessed via the Autism Quotient (AQ; [92]). This self-report questionnaire assesses five different domains relevant to autistic characteristics (attention switching, attention to detail, communication, social skill and imagination). Participants are instructed to respond to 50 items with one of four responses: ‘definitely agree’, ‘slightly agree’, ‘slightly disagree’ and ‘definitely disagree’. These responses are scored using a binary system, where endorsing an autistic trait (either mildly or strongly) is scored as 1, and not endorsing an autistic trait (either mildly or strongly) is scored as 0. As such, scores on the AQ range from 0 to 50, with higher scores representing higher levels of autistic traits. The English [93], French [94], and Japanese [95] versions of the AQ have strong psychometric properties.

#### 4.2.7. Alexithymic traits

The alexithymic traits of participants will be measured via the 20-item Toronto Alexithymia Scale [96]. This self-report questionnaire comprises 20 items rated on a five-point Likert scale (from 1, strongly disagree, to 5, strongly agree). Total scores on the TAS-20 range from 20 to 100, with higher scores representing higher levels of alexithymic traits. The TAS-20 is the most popular tool for assessing alexithymia and the English [96, 97], French [98], and Japanese [99] versions have good psychometric properties.

#### 4.2.8. Translation of relevant questionnaires

Many of the questionnaires used in this study (e.g., the Depression, Anxiety and Stress Scale, the Autism Quotient, the Toronto Alexithymia Scale) have already been translated into the target languages (English, French, Japanese). However, other questionnaires (e.g., the Autism Acceptance Questions and the CAT-Q) are yet to be translated. We will follow the recommendations outlined in Gorecki et al., [100] for conducting translations for cross-cultural research. As such, we will use the forward-backward method of translation that has been used by other researchers in this field (e.g., [69]). The psychometric properties of the translated questionnaires will be assessed and reported in the manuscript (as was the case in Someki et al. [69]). More specifically, following the procedures outlined in Cage et al., [34] and Hull et al., [48], we will assess a) the internal consistency of the translated questionnaires via item analyses, and b) the factor structure of the translated questionnaires via principal component analyses.

## 5. Analysis plan

*You may describe one or more confirmatory analysis in this preregistration. Please remember that all analyses specified below must be reported in the final article, and any additional analyses must be noted as exploratory or hypothesis generating*.

*A confirmatory analysis plan must state up front which variables are predictors (independent) and which are the outcomes (dependent), otherwise it is an exploratory analysis. You are allowed to describe any exploratory work here, but a clear confirmatory analysis is required*.

### 5.1. Statistical models (required)

First, in our regression analyses, we will include data for all participants that completed the study in one group (collapsing across countries) to explore relationships in a large and cross-cultural sample of autistic adults. For these four regression analyses, we will use a p < .0125 threshold to establish whether to reject the null hypothesis (corrected for multiple testing). Then, we will separate participants into groups based on their country of residence and compare participants’ experiences of autism acceptance, camouflaging, and levels of depression, anxiety and stress across these groups.

#### 5.1.1. Replicating the regression analyses from previous research

Following the procedures from Cage et al., [37], we will complete two hierarchical regression analyses with depression and stress as the outcome variables respectively. Blockwise entry will be used to analyse the data: in step one, age, age of diagnosis, gender and the other DASS subscale scores will be entered. In the second step, the two types of acceptance (external sources and personal) will be added.

In line with the procedures outlined in Perry et al., [59], we will complete a multiple regression analysis with camouflaging as the dependent variable, and autism acceptance, age, gender, age of diagnosis and autistic tratis as the independent variables.

As in Hull et al., [58], we will complete a hierarchical regression analysis with anxiety as the dependent variable. In step one, age and autistic traits will be added into the model. In step two, camouflaging will be entered into the model.

#### 5.1.2. Comparing autism acceptance, camouflaging and mental health across cultures

Finally, in order to compare participants’ experiences of autism acceptance, camouflaging, and levels of depression, anxiety and stress across countries, we will use a multivariate analysis of variance (MANOVA). This MANOVA will have the between-subjects factor *country* and the dependent variables *external acceptance*, *personal acceptance*, *depression*, *anxiety*, *stress*, and *camouflaging*. This will facilitate exploration of group differences in the levels of autism acceptance, camouflaging and mental health difficulties.

### 5.2. Transformations (optional)

In the instance that the data violates any assumptions of the planned statistical analyses, standard transformations will be completed such that the data can be analysed with parametric tests. If assumptions remain unmet, non-parametric analyses will be run. In any analyses that investigate the contribution of multiple predictor variables to a dependent variable, the predictor variables will be z-scored to facilitate comparisons.

### 5.3. Inference criteria (optional)

For our regression analyses, to account for the four tests we will use a p < .0125 significance threshold to determine whether to accept or reject the null hypothesis. For our MANOVA, we will use a p = .05 significance threshold.

In our analyses, Bayes Factors (BF_01_) may be reported, which provide a ratio of the likelihood for the observed data under the null hypothesis compared to the alternative hypothesis [101]. For all Bayesian analyses, we will follow the classification scheme in JASP outlined by Lee and Wagenmakers [102]: BF_10_ values between one and three will be considered as weak evidence, between three and ten as moderate evidence and greater than ten as strong evidence for the alternative hypothesis.

### 5.4. Data exclusion (optional)

We will exclude any participants that state that they do not have a diagnosis of autism from our analyses. In order to represent the diverse experiences of autistic individuals, we aim to include as much participant data as possible in our analyses. However, we will use Mahalanobis Distance to determine multivariate outliers in our data. Outliers that persist after any necessary transformations and centering (e.g., z-scoring) will be excluded on a case-by-case basis.

### 5.5. Missing data (optional)

We will use multiple imputation to handle missing data. This statistical technique is a relatively flexible, general purpose approach to dealing with missing data [103].

### 5.6. Exploratory analysis (optional)

We will explore the relationships between our dependent variables (external acceptance, personal acceptance, depression, anxiety, stress and camouflaging) and various individual differences (e.g., autistic traits, alexithymic traits, gender, level of education, age of diagnosis, income etc.). For continuous variables, we will run simple (Bonferroni-corrected) correlations between our dependent variables and demographic factors (e.g., age, autistic traits). For categorical variables, we will use Bonferroni-corrected independent samples t-tests to compare levels of autism acceptance, camouflaging and mental health difficulties across the demographic factor (e.g., gender). For example, as gender is a categorical variable, we will use Bonferroni-corrected independent samples t-tests to compare levels of acceptance, camouflaging and mental health across genders. Conducting these analyses will allow us to verify whether any individual differences are associated with any of our dependent variables as has been found previously in some instances (e.g., camouflaging and gender; [41]).

Then, we will conduct our MANOVA again whilst controlling for any of the individual differences that are associated with our dependent variables or which differ significantly between groups. This will allow us to determine whether there are cultural differences in autism acceptance, camouflaging and mental health that go beyond these specific individual differences.

Moreover, in order to investigate whether camouflaging mediates any relationships we identify between autism acceptance and mental health outcomes, we will complete mediation analyses. There are several methods used to test the statistical significance of mediated effects [104–106]. In the present study, we will use structural equation modelling (SEM), a powerful statistical technique used to infer causal relationships between variables, for our mediation analyses. We decided to use SEM over standard regression methods (as outlined by Baron and Kenny; [107]) as this technique is regarded as “a more appropriate inference framework for mediation analyses” (see [108]). In addition, we will use bias-corrected bootstrapping to create confidence intervals as there is a consensus that it is the most powerful method for testing mediated effects [104, 109–112]. Whilst we believe that there will be sufficient statistical power to detect an effect, we may supplement this analysis with Bayesian mediation analyses if it appears that we are underpowered.

In addition, we will calculate standardised residuals for the relationships between autism acceptance and mental health, autism acceptance and camouflaging, and camouflaging and mental health, for all participants. Then we will complete an exploratory MANOVAs in which we will compare the standardised residuals across countries. This will allow us to compare the strength of these relationships in each of the countries by comparing the levels of unexplained variance following regression analyses. This is useful because the strengths of these relationships could, in principle, vary across our countries. For example, it could be that in collectivist, relative to individualistic, cultures, camouflaging accounts for a smaller portion of variance in mental health as hiding any traits that deviate from the norm is common (and therefore may not be detrimental to mental health).

Finally, in order to compare the strength of the relationships between our dependent variables across genders, we will complete an additional exploratory MANOVA using the standardised residuals discussed above. This is useful because the strength of the relationships between our dependent variables could vary across genders: it could be the case, for instance, that there is a stronger relationship between autism acceptance and camouflaging in autistic women than autistic men, thus explaining the higher rates of camouflaging displayed by autistic women [40–48]. In addition, it could be that there is a weaker relationship between camouflaging and mental health difficulties in autistic men since they more frequently report positive consequences of camouflaging [38].

## 6. Other

### 6.1. Other (optional)

Please note that the authors consulted an autism consultancy group (Birmingham Psychology Autism Research Team Consultancy Committee) and the global U21 Autism Research Network when designing the study in order to confirm that a) the aims of the study were in line with community priorities, b) that the questionnaires were clear and accessible to autistic participants and c) that the aims and methodology were in line with norms for research conduct in each participating country.
